# Analysis of Inflammatory Mediators in Prediabetes and Newly Diagnosed Type 2 Diabetes Patients

**DOI:** 10.1155/2016/7965317

**Published:** 2016-07-05

**Authors:** Zhen Wang, Xu-Hui Shen, Wen-Ming Feng, Guo-fen Ye, Wei Qiu, Bo Li

**Affiliations:** ^1^Department of Clinical Medicine, School of Nursing & Medicine, Huzhou University, Huzhou, Zhejiang 313000, China; ^2^Department of Nursing Medicine, School of Nursing & Medicine, Huzhou University, Huzhou, Zhejiang 313000, China; ^3^Surgery Department, Huzhou Wu Xing People's Hospital, Huzhou, Zhejiang 313008, China; ^4^Physical Examination Center, Zhebei Clinical Medicine Hospital, Huzhou University, Huzhou, Zhejiang 313000, China; ^5^Endocrinology Department, Zhebei Clinical Medicine Hospital, Huzhou University, Huzhou, Zhejiang 313000, China; ^6^Huanzhu Street Community Health Center, Huzhou, Zhejiang 313000, China

## Abstract

This study evaluated the inflammatory markers in prediabetes and newly diagnosed type 2 diabetes mellitus (T2DM). Inflammatory markers levels were analyzed using one-way analysis of covariance and the association with prediabetes or T2DM risks was examined by logistic regression models. Our data showed increased levels of hypersensitivity C-reactive protein (hs-CRP), interleukin (IL-4), IL-10, and tryptase in prediabetes subjects and hs-CRP, immunoglobulin E (IgE), IL-4, and IL-10 in T2DM subjects. We concluded that Hs-CRP, IgE, IL-4, IL-10, and tryptase were positively associated with prediabetes or T2DM. Further large prospective studies are warranted to assess a temporal relation between inflammatory biomarkers and incidence of prediabetes or T2DM and its associated chronic diseases.

## 1. Introduction

Type 2 diabetes mellitus (T2DM) is a complex disease in which both genetic and environmental factors interact in determining impaired *β*-cell insulin secretion and peripheral insulin resistance [1–3]. T2DM is also a metabolic disorder between pro- and anti-inflammatory characterized by chronic hyperglycemia and increased or decreased levels of circulating cytokines [4]. The rise in the proinflammatory cytokines (e.g., interleukin- (IL-) 1, IL-6, tumor necrosis factor- (TNF-) *α*, C-reactive protein (CRP), transforming growth factor- (TGF-) *β*, and leptin) or the fall in anti-inflammatory cytokines (e.g., interleukin-1 receptor antagonist (IL-1Ra), IL-4, IL-10, IL-13, and adiponectin) is the essential step in glucotoxicity and lipotoxicity induced mitochondrial injury, oxidative stress, and beta cell apoptosis in T2DM [2–4]. These pro- and anti-inflammatory cytokines can enhance insulin resistance directly in adipocytes, muscle, and hepatic cells, leading to systemic disruption of insulin sensitivity and impaired glucose homeostasis [5].

Many single nucleotide polymorphisms (SNPs) in various genes including those of pro- and anti-inflammatory cytokines have been reported as a risk for T2DM [1–3]. But not all SNPs have been confirmed by unifying results in different studies and wide variations have been reported in various ethnic groups [2–4]. Genetic polymorphisms of C-reactive protein (CRP) and their association with prediabetes and T2DM have been widely studied [5]. IL-6 was regarded as a kind of pro- and anti-inflammatory factor; one of the common polymorphisms in the IL-6 gene promoter (C-174G) was considered as risk factors for T2DM development [2]. IL-10 is an anti-inflammatory cytokine protecting against T2DM and inflammation; several variants in the IL-10 gene promoter region have been identified and showed the association with the development of T2DM [3, 4]. It is reported that TNF-*α* is a possible mediator of insulin resistance and diabetes since it inhibits insulin signaling and impairs its secretion [2–4]. One of the SNPs in TNF-*α* gene showed a twofold increase in transcriptional activity and an association of TNF-*α* SNPs with T2DM [2]. Genetic variants in some inflammatory factors associated with T2DM may provide a rationale for further studying their roles as biomarkers for disease early risk prediction and therapeutic targets for T2DM and related complications.

Although accumulating evidences support the pathological role of inflammatory cytokines in T2DM, most studies only focused on a few specific inflammatory factors and were done in relatively small population groups in a particular ethnic group. In our previous studies, we have explored the associations between few several inflammatory cytokines (e.g., CRP, immunoglobulin E (IgE), chymase, and tryptase) with prediabetes and T2DM and concluded that IgE and CRP are risk factors of prediabetes and T2DM only based on a small sample of cross-sectional study design [6, 7].

In this study, we extend the analysis of the role of pro- and anti-inflammatory cytokines in prediabetes and newly diagnosed T2DM based on a larger sample of cross-sectional study design, by measuring the levels of several proinflammatory cytokines (e.g., IgE, hs-CRP, IL-6, TNF-*α*, and tryptase) and anti-inflammatory cytokines (e.g., IL-4, IL-10, and forkhead/winged helix transcription factor 3+ (Foxp3+)) in prediabetes and T2DM, and correlate them with other laboratory and clinical biochemical indicators. Our findings will lend support to the hypothesis that some specific inflammatory factors may play an etiological role in the pathogenesis of T2DM and also will offer new insights into the potential clinical value of these inflammatory factors as biomarkers for disease early risk prediction and therapeutic targets for T2DM and related complications.

## 2. Materials and Methods

### 2.1. Study Population

The study is part of the Diabetes Intervention Project (DIP) started in 2012 from School of Nursing and Medicine, Huzhou University, Zhejiang, China. From March to December 2012, a total of 7054 rural residents aged 50–75 years from eight rural communities in the city of Huzhou participated in physical examination. Based on the criteria of fasting plasma glucose (FPG) <5.6 mmol/L, 898 (12.73%) were classified into prediabetes. During July to August 2013, after excluding subjects with known DM or receiving hypoglycaemic medications or with cardiovascular disease, cerebrovascular disease, malignant disease, chronic liver disease, or kidney failure, or those under medications, 825 cases of 898 prediabetes subjects and another 300 randomized sampling normal glucose subjects were invited for FPG, 2-hour oral glucose tolerance test (2 h OGTT), and haemoglobin A1c (HbA1c) tests as part of the prediabetes and T2DM screening. But only 560 subjects would participate in this study, according to the following clinical criteria based on FPG, 2 h OGTT, and HbA1c test: 219 (39.11%) had normal glucose tolerance (NGT), 215 (38.39%) were diagnosed as prediabetes subjects, and 126 (22.5%) were diagnosed as T2DM subjects. This study was approved by the Huzhou City Ethics Committee and all subjects gave written, informed consent prior to participating in the study.

### 2.2. Data Collection

Trained staff interviewed participants using a self-designed questionnaire to obtain information on demographic characteristics and anthropometric and lifestyle variables. Physical activity assessments were performed using self-reported Total Energy Expenditure Questionnaire (TEEQ), a nine-step scale where every step was assigned a fixed value in terms of multiple of Metabolic Energy Turnover (MET) [8]. Anthropometric measurements (body height and weight, waist circumference (WC), hip circumference, and blood pressure) were attained at the initial screening visit. Two sitting blood pressure measurements were taken for each participant using a mercury sphygmomanometer according to a standard protocol. The mean of these two blood pressure measurements was used in the data analysis.

The biochemical parameters, including FPG, 2 h OGTT, HbA1c, fasting insulin, plasma total cholesterol (TC), triglyceride (TG), low-density lipoprotein cholesterol (LDL-c), high-density lipoprotein cholesterol (HDL-c), hs-CRP, and IgE, were measured in the Clinical Biochemistry Unit of Huzhou First Hospital, a teaching hospital of Huzhou University. The cytokine concentrations, including IL-6, TNF-*α*, tryptase, IL-4, IL-10, and Foxp3+, were measured in patients sera using commercially available ELISA double antibody sandwich method assays (R&D Systems), performed according to the manufacturer's instructions. Detection kits were produced by Wuhan Gene Biotech Co., Ltd.

### 2.3. Clinical Criteria

Diabetes and prediabetes were grouped according to American Diabetes Association 2010 (ADA 2010) criteria or HbA1c-based diagnosed criteria [9, 10]. Diabetes was classified with a fasting plasma glucose (FPG) ≥ 7.0 mmol/L or 2 h OGTT ≥ 11.1 mmol/L or HbA1c ≥ 6.5%, whereas prediabetes was defined as FPG ≥ 5.6 and <7.0 mmol/L or 2 h OGTT ≥ 7.8 and <11.1 mmol/L or HbA1c ≥ 6.0% and HbA1c < 6.5%. Subjects were classified as having a normal glucose profile if FPG < 5.6 mmol/L and 2 h OGTT < 7.8 mmol/L or HbA1c < 6.0%.

### 2.4. Statistical Analysis

The mean and standard deviation (mean ± SD) of continuous and normal distributional variables and median and quartile range of continuous but skewed distributional variables were used.

Body mass index (BMI) was calculated as weight in kilograms divided by the square of the height in meters (kg/m^2^). Waist-to-hip ratio (WHR) was calculated as waist divided by hip circumference. Homeostasis model assessment-insulin resistance (HOMA-IR) was calculated as value of FPG × value of fasting insulin/22.5 and homeostasis model assessment-*β* cell function (HOMA-*β*) was calculated as 20 × value of fasting insulin/(FPG-3.5).

Based on China 2006 Blood Pressure Control Criteria and China Prevention and Treatment Classification Recommendation on Dyslipidemia [11], hypertension was defined as systolic blood pressure (SBP) and/or diastolic blood pressure (DBP) ≥ 140/90 mmHg or as receiving blood pressure lowering medications; high TG was defined as a fasting plasma TG ≥ 1.70 mmol/L, low HDL-C as a fasting HDL-C ≤ 0.9 mmol/L, high TC as TC ≥ 5.72 mmol/L, and low LDL-C as a fasting LDL-C ≤ 3.64 mmol/L. Based on China Obesity Task Group Recommendation [12], overweight was classified when a body mass index (BMI) ≥ 24 kg/m^2^ and obesity was classified when a body mass index (BMI) ≥ 29 kg/m^2^.

In analyzing the relationships of inflammatory cytokines to prediabetes or T2DM, IgE was classified as normal/abnormal according to upper quartiles (P75 = 34.0 IU/L); CRP was classified as normal/abnormal according to upper quartiles (P75 = 1.0 mg/L); tryptase was classified as normal/abnormal according to upper quartiles (P75 = 1699.94 ng/mL) and TNF-*α* was classified as normal/abnormal according to upper quartiles (P75 = 325.14 pg/mL); IL-4 was classified as normal/abnormal according to upper quartiles (P75 = 323.91 ng/L); IL-10 was classified as normal/abnormal according to upper quartiles (P75 = 256.22 ng/L); FOXP3+ was classified as normal/abnormal according to upper quartiles (P75 = 411.17 pg/mL).

One-way analysis of covariance was used to test for differences in continuous distributional variables between three groups. The nonparametric Kruskal-Wallis test was used to test for differences in continuous but skewed distributional variables between two groups, and *χ* ^2^ test was used to test differences in proportions between three groups.

Spearman correlation coefficients were calculated to evaluate associations between inflammatory cytokines and the traditional cardiovascular factors.

Binary logistic regression model was used to estimate the odds ratios (ORs) and 95% CIs for prediabetes and T2DM according to dichotomy of inflammatory biomarkers concentration, using the lower values as the referent category. Considering the potential confounding factors, we applied 10 models to assess the association between biomarkers and prediabetes or T2DM, including unadjusted mode, age and sex-adjusted model, multivariable (age, sex, BMI, WHR, SBP, DBP, TC, TG, level of physical activity, dietary intake, alcohol intake, smoking status, presence or absence of family history of diabetes, hypertension, heart disease, stroke, and hypercholesterolemia) adjusted model, and multivariable combined with inflammatory biomarkers mutually adjusted model.

All statistical analysis was conducted using SPSS statistical software (version 19.0).

## 3. Results

The descriptive characteristics of 560 study participants were presented separately for participants with normal glucose tolerance, prediabetes, and T2DM (Table 1). A total of 215 were prediabetes, 126 were T2DM, and 219 were normal glucose subjects. Overall, except smoking status, alcohol use, disease family history, physical activity level, and dietary intake, all traditional vascular risk factors were worse in the PDG or the T2DMG patients than NGG subjects. Median levels of IgE in T2DMG subjects (40 IU/L) were significantly higher compared with NGG subjects (18 IU/L) (*P* < 0.05) and with PDG subjects (20 IU/L) (*P* < 0.05). Median levels of CRP in PDG subjects (0.8 mg/L) or T2DMG subjects (1.5 mg/L) were significantly higher compared with NGG subjects (0.5 mg/L) (*P* < 0.05). Mean levels of tryptase in PDG subjects (mean ± SD: 1545.36 ± 291.45 ng/mL) or T2DMG subjects (mean ± SD: 1524.96 ± 286.65 ng/mL) were significantly higher compared with NGG subjects (mean ± SD: 1481.21 ± 271.44 ng/mL) (*P* < 0.05). Mean levels of IL-4 in PDG subjects (mean ± SD: 303.70 ± 56.84 ng/L) or T2DMG subjects (mean ± SD: 304.45 ± 55.87 ng/L) were significantly higher compared with NGG subjects (mean ± SD: 283.45 ± 52.50 ng/L) (*P* < 0.05). Mean levels of IL-10 in PDG subjects (mean ± SD: 244.03 ± 53.34 ng/L) or T2DMG subjects (mean ± SD: 265.04 ± 40.42 ng/L) were significantly higher compared with NGG subjects (mean ± SD: 229.23 ± 46.54 ng/L) (*P* < 0.05). But there were no significant differences in IL-6, TNF-*α*, and Foxp3+ (Table 1).

Spearman correlation analysis between inflammatory cytokines and the traditional cardiovascular factors indicated that whether, in NGG, the PDG, or the T2DMG, there were significant relationships between most of the traditional cardiovascular factors and between most of inflammatory cytokines. However, there were seldom significant relationships between the traditional cardiovascular factors and inflammatory cytokines (Tables 2 [Table tab3]–4; Tables  S1–S6) (in Supplementary Material available online at http://dx.doi.org/10.1155/2016/7965317).

After adjusting confounding factors by multivariable combined with inflammatory biomarkers mutually adjusted model, plasma IgE was associated with T2DM compared with NGG (OR (odds ratio): 3.46 (1.92–6.23, 95% CI), *P* < 0.001) or compared with PDG (OR: 2.57 (1.54–4.30, 95% CI), *P* < 0.001); plasma CRP was associated with PDG (OR: 2.30 (1.46–3.62, 95% CI), *P* < 0.001) or T2DM (OR: 16.24 (7.99–33.02, 95% CI), *P* < 0.001) compared with NGG or T2DM (OR: 5.93 (3.29–10.72, 95% CI), *P* < 0.001) compared with PDG; serum IL-4 was associated with PDG (OR: 1.68 (1.05–2.69, 95% CI), *P* = 0.031) or T2DM (OR: 1.94 (1.10–3.53, 95% CI), *P* = 0.023) compared with NGG; serum IL-10 was associated with PDG (OR: 2.13 (1.33–3.41, 95% CI), *P* = 0.002) or T2DM (OR: 8.62 (4.50–16.49, 95% CI), *P* < 0.001) compared with NGG or T2DM (OR: 3.04 (1.79–5.13, 95% CI), *P* < 0.001) compared with PDG (Table 5). Serum tryptase was associated with prediabetes (OR: 1.53 (1.01–2.32, 95% CI), *P* = 0.044) before adjustment and (OR: 1.63 (1.02–2.61, 95% CI), *P* = 0.041) after adjustment by multivariable + IL-4, compared with NGG. Serum IL-6, TNF-*α*, and Foxp3+ were not associated with PDG or T2DM (Table 5).

## 4. Discussion

Our data showed increased levels of CRP, IL-4, IL-10, and tryptase in prediabetes subjects and increased levels of CRP, IL-4, and IL-10 in T2DM subjects compared with normal glucose subjects. Not all inflammatory cytokines in our study were in agreement with previous findings.

With the associations between inflammatory cytokines (e.g., IL-6, TNF-*α*, and hs-CRP) and insulin resistance, prediabetes or T2DM had been widely researched [13–21]. Most studies found that subjects with insulin resistance, prediabetes, or T2DM had increased levels of IL-6, TNF-*α*, and hs-CRP [13–16]. A study reported that high levels of inflammatory cytokines appeared in early stage of T2DM and were capable of predicting the development of type 2 diabetes through diminishing insulin sensitivity [14]. But a recent study showed decreased levels of IL-6 and TNF-*α* in T2DM compared to healthy controls [17]. The author in this paper interpreted that this discrepancy could be attributed to duration of the diseases, small sample size, and the differences in age and sex of the studied groups [17]. Elevated plasma levels of hs-CRP in prediabetes and T2DM subjects have been demonstrated in this paper (Tables 1 [Table tab2] [Table tab3] [Table tab4] and 5), our previous studies, and other studies [6, 7, 14–21]. Our data showed that mean levels of TNF-*α* or IL-6 in PDG or T2DMG subjects were higher but insignificant compared with NGG subjects (Tables 1 and 5), which could be explained by the differences of some incompletely measured residual confounding factors or inflammation-related diseases (e.g., rheumatoid arthritis (RA) or osteoarthritis (OA)) or allergies in the studied groups.

IgE stimulation during allergic reactions and infections is the natural defense mechanism. It also plays a crucial role in the pathophysiology of T2DM [6, 7]. This current study demonstrated that plasma IgE levels strongly correlated with T2DM (Table 5), which was in agreement with our previous studies [6, 7]. Further large population-based prospective studies are warranted to assess the role of IgE in T2DM.

IL-4 was major anti-inflammatory cytokine and had been proposed to play a crucial role in the pathophysiology of T2DM, and several candidate genes have been identified [22]. Our data showed that serum IL-4 was associated with PDG or T2DM (Tables 1 and 5).

IL-10 was anti-inflammatory cytokine and has been shown to improve impaired insulin signaling [23], prevent the development of IL-6 [24], inhibit NADPH oxidase [25], prevent pancreatic beta cell destruction [26], and play an important role in modulation of cardiovascular insulin resistance [23–26]. Two studies indicated that low IL-10 level was associated with high HbA1c and serum IL-10 level may be one of the predictors of glycemia [27, 28]. Increased level of IL-10 in prediabetes and T2DM subjects in our study was in disagreement with these findings [2, 26–28], which could be explained by high levels of inflammatory cytokines appearing in early stage of T2DM [14, 17].

T regulatory (Treg) cells are a unique population of T-cells which play a crucial role in the maintenance of self-tolerance and suppression of potentially inflammatory T-cells [29]. Chronic low-grade inflammation in obesity and impaired insulin sensitivity has been associated with fewer Tregs in adipose tissue [29–31]. Our study did not prove the associations between FOXP3+ with the PDS or T2DM (Tables 1 and 5).

Mast cell tryptase is an important protease that has been implicated in cardiovascular diseases [32–34]. Our previous study did not demonstrate the association between plasma tryptase with prediabetes or T2DM [6, 7]. But in this paper, our data indicated that tryptase was mildly associated with prediabetes (OR: 1.63 (1.02–2.61, 95% CI), *P* = 0.041) after adjustment by multivariable + IL-4, compared with NGG, which could be explained for the synergistic effect of IL-4.

Our data also showed that there were significant relationships between most of the traditional cardiovascular factors and between most of inflammatory cytokines. However, there were seldom significant relationships between the traditional cardiovascular factors and inflammatory cytokines (Tables 2 [Table tab3]–4; Tables  S1–S6), which indicated that complex interactions could exist between these inflammatory cytokines or between cardiovascular factors [1–3, 20, 21].

Results of our study extend other evidences linking inflammatory cytokines to insulin resistance and risk of diabetes based on a larger sample of cross-sectional study design. Our findings further lend support to the hypothesis that some specific inflammatory factors may play an important role in the pathogenesis of T2DM and also offer new insights into the potential clinical value of these inflammatory factors as biomarkers for T2DM early prediction and treatment [13–17].

Our study has several limitations. We assessed the associations between few inflammatory cytokines and prediabetes and T2DM by means of plasma or serum biomarker only based on cross-sectional study design and cannot demonstrate that altered plasma or serum levels of inflammatory biomarkers are predictors of incident diabetes by large prospective cohort study. Our comprehensive assessment of diabetes risk factors allowed statistical control for important confounding factors in the pathogenesis of diabetes, but residual confounding could remain in the analysis. In particular, we did not consider potential inflammation-related diseases, such as RA or OA, which widely exist in the senior, or allergies which are also very common among individuals, and these unmeasured residual confounding factors might result in discrepancies.

In conclusion, circulating inflammatory mediators, hs-CRP, IgE, IL-4, IL-10, and tryptase, were positively associated with prediabetes or T2DM. Further large prospective studies are warranted to assess a temporal relation between baseline levels of inflammatory biomarkers and incidence of prediabetes or T2DM and its associated chronic diseases.

## Supplementary Material

Tables S1-S6 described the spearman correlation between inflammatory cytokines and the traditional cardiovascular factors and indicated that whether in the Pre-diabetes group or the type 2 diabetes mellitus group, there were significant relationships between most of the traditional cardiovascular factors and between most of inflammatory cytokines. However, there were seldom significant relationships between the traditional cardiovascular factors and inflammatory cytokines (Tables S1-S6).

## Figures and Tables

**Table 1:** Baseline characteristics of 560 subjects with different glucose status grouped according to fasting, postload glucose levels, and HbA1c.

Variable	NGG (*n* = 219)	PDG (*n* = 215)	T2DMG (*n* = 126)	*P* value
Age (years)^*∗*^	58.13 ± 6.37	59.31 ± 6.86	59.37 ± 7.07	0.122
Sex (%, male)	47.0	40.5	40.5	0.310
BMI (kg/m^2^)^*∗*^	23.58 ± 3.17	25.11 ± 3.51^†^	24.55 ± 3.15^‡^	**<0.001**
WC (cm)^*∗*^	79.92 ± 9.19	85.00 ± 9.89^†^	83.85 ± 8.90^‡^	**<0.001**
WHR^*∗*^	0.84 ± 0.06	0.87 ± 0.05^†^	0.87 ± 0.06^‡^	**<0.001**
Smoking status (%)				0.071
Current	63.9	28.8	7.3	—
Never	73.0	19.1	7.9	—
Past	63.0	31.7	7.9	—
Alcohol use (in the past 12 months) (%, drinker)	25.1	17.2	22.2	0.130
History of diabetes (%, yes)	6.8	12.6	29.4	**<0.001**
History of myocardial infarction (%, yes)	0.9	0.9	2.4	0.493
History of high blood pressure (%, yes)	15.1	14.0	18.3	0.689
History of stroke (%, yes)	4.6	5.6	4.8	0.731
History of dyslipidemia (%, yes)	8.7	6.5	13.5	0.360
Physical activity (MET/week)^*∗*^	440.47 ± 146.79	447.92 ± 151.39	427.49 ± 152.15	0.480
Dietary intake (%, excess energy intake)	74.9	72.1	73.0	0.800
Carbohydrate intake (%)				0.740
<55% of total energy intake	56.6	54.9	50.8	—
55–65% of total energy intake	24.2	24.2	23.8	—
>65% of total energy intake	19.2	20.9	25.4	—
Fat intake (%)				**0.005**
<20% of total energy intake	11.9	20.0	23.8	—
20–25% of total energy intake	41.1	38.1	46.8	—
>25% of total energy intake	47.0	49.1	29.4	—
Protein intake (%)				0.529
<60% of total energy intake	16.9	11.2	12.7	
60–65% of total energy intake	29.7	31.6	31.7	
>65% of total energy intake	53.4	57.2	55.6	
FPG (mmol/L)^*∗∗*^	5.17 (4.94–5.34)	5.97 (5.74–6.20)^†^	7.72 (7.35–8.89)^‡,§^	**<0.001**
2 h OGTT (mmol/L)^*∗*^	5.35 ± 1.08	7.64 ± 1.48^†^	12.16 ± 1.55^‡,§^	**<0.001**
HbA1c (%)^*∗*^	5.06 ± 0.80	5.20 ± 1.03	6.64 ± 1.64^‡,§^	**<0.001**
Fasting insulin (mU/L)^*∗∗*^	7.0 (6.55–7.36)	7.36 (6.89–8.70)^†^	10.10 (8.54–13.7)^‡,§^	**<0.001**
HOMA-IR^*∗∗*^	1.60 (1.42–1.73)	2.04 (1.81–2.32)^†^	3.64 (2.93–4.58)^‡,§^	**<0.001**
HOMA-*β* ^*∗∗*^	84.14 (72.72–99.79)	60.27 (52.46–70.64)^†^	48.67 (32.96–66.35)^‡,§^	**<0.001**
SBP (mmHg)^*∗*^	121.74 ± 20.01	130.94 ± 23.61^†^	130.49 ± 18.90^‡^	**<0.001**
DBP (mmHg)^*∗*^	76.46 ± 9.97	80.53 ± 11.29^†^	77.83 ± 8.31^§^	**<0.001**
TC (mmol/L)^*∗*^	4.85 ± 0.84	5.00 ± 0.90^†^	5.24 ± 1.12^‡^	**0.001**
TG (mmol/L)^*∗∗*^	1.33 (1.04–1.94)	1.72 (1.30–2.44)^†^	1.95 (1.31–3.17)^‡^	**<0.001**
HDL-c (mmol/L)^*∗*^	1.29 ± 0.32	1.20 ± 0.30^†^	1.16 ± 0.28^‡^	**<0.001**
LDL-c (mmol/L)^*∗*^	2.85 ± 0.75	2.80 ± 0.92	2.90 ± 1.04	0.613
IgE (IU/L)^*∗∗*^	18 (7–34)	20 (7–42.5)	40 (16–64)^‡,§^	**<0.001**
hs-CRP (mg/L)^*∗∗*^	0.5 (0.3–1.0)	0.8 (0.4–2.0)^†^	1.50 (1.00–2.40)^‡,§^	**<0.001**
IL-6 (ng/L)^*∗*^	58.56 ± 12.66	60.92 ± 12.82	60.39 ± 10.28	0.117
TNF-*α* (pg/mL)^*∗*^	284.94 ± 61.17	290.13 ± 68.22	295.37 ± 57.25	0.326
Tryptase (ng/mL)^*∗*^	1481.21 ± 271.44	1545.36 ± 291.45^†^	1524.96 ± 286.65^‡^	**0.036**
IL-4 (ng/L)^*∗*^	283.45 ± 52.50	303.70 ± 56.84^†^	304.45 ± 55.87^‡^	**<0.001**
IL-10 (ng/L)^*∗*^	229.23 ± 46.54	244.03 ± 53.34^†^	265.04 ± 40.42^‡,§^	**<0.001**
Foxp3+ (pg/mL)^*∗*^	364.22 ± 72.27	367.26 ± 24.46	356.18 ± 83.12	0.418

T2DM: type 2 diabetes mellitus; NGG: normal glucose group; PDG: prediabetes group; T2DMG: T2DM group; BMI: body mass index; WC: waist circumference; WHR: waist hip ratio; HOMA-IR: homeostasis model assessment-insulin resistance; HOMA-*β*: homeostasis model assessment-*β* cell function; FPG: fasting plasma glucose; 2 h OGTT: 2-hour oral glucose tolerance test; HbA1c: haemoglobin A1c; SBP: systolic blood pressure; DBP: diastolic blood pressure; TC: total cholesterol; TG: triglyceride; LDL-c: low-density lipoprotein cholesterol; HDL-c: high-density lipoprotein cholesterol; IgE: immunoglobulin E; hs-CRP: hypersensitivity C-reactive protein; TNF-*α*: tumor necrosis factor; IL-6: interleukin-6; IL-4: interleukin-4; IL-10: interleukin-10; Foxp3+: forkhead/winged helix transcription factor 3+.

^*∗*^Variable is described using mean and standard deviation. ^*∗∗*^Variable is described using median and interquartile range. ^†^ *P* < 0.05, PDG compared with the NGG group; ^‡^ *P* < 0.05, DMG compared with the NGG group. ^§^ *P* < 0.05, T2DMG compared with the PDG group.

**Table 2 tab2:** Spearman correlation coefficients between cardiovascular factors in normal glucose group.

	TC	TG	HDL_C	LDL_c	HOMA-IR	HOMA-*β*	BMI	WHR	SBP
TC	1								
TG	0.321^*∗∗*^	1							
HDL_C	−0.298^*∗∗*^	−.0363^*∗∗*^	1						
LDL	0.878^*∗∗*^	0.098	0.107	1			·		
HOMA-IR	0.062	0.042	−0.120	0.036	1		·		·
HOMA-*β*	−0.094	−0.137^*∗*^	0.096	−0.102	−0.140^*∗*^	1			
BMI	0.203^*∗∗*^	0.374^*∗∗*^	−0.279^*∗∗*^	0.209^*∗∗*^	0.062	−0.213^*∗∗*^	1		
WHR	0.029	0.363^*∗∗*^	−0.386^*∗∗*^	0.054	0.069	−0.112	0.552^*∗∗*^	1	·
SBP	0.184^*∗∗*^	0.144^*∗*^	−0.014	0.161^*∗*^	0.053	−0.124	0.284^*∗∗*^	0.252^*∗∗*^	1
DBP	0.166^*∗*^	0.220^*∗∗*^	−0.105	0.148^*∗*^	0.057	−0.074	0.406^*∗∗*^	0.335^*∗∗*^	0.716^*∗∗*^

TC: total cholesterol; TG: triglyceride; LDL-c: low-density lipoprotein cholesterol; HDL-c: high-density lipoprotein cholesterol; HOMA-IR: homeostasis model assessment-insulin resistance; HOMA-*β*: homeostasis model assessment-*β* cell function; BMI: body mass index; WHR: waist hip ratio; SBP: systolic blood pressure; DBP: diastolic blood pressure.

^*∗*^Data on all subjects without missing values for all of these variables. ^*∗*^ *P* < 0.05. ^*∗∗*^ *P* < 0.01.

**Table 3 tab3:** Spearman correlation coefficients between inflammatory markers in normal glucose group.

	IgE	CRP	IL-4	IL-6	IL-10	Foxp3+	Tryptase
IgE	1						
CRP	0.117	1					
IL-4	0.059	−0.174^*∗∗*^	1	·	·		
IL-6	0.026	0.06	0.303^*∗∗*^	1			
IL-10	0.087	−0.012	0.272^*∗∗*^	0.259^*∗∗*^	1		
Foxp3+	0.071	−0.066	0.211^*∗∗*^	0.245^*∗∗*^	0.430^*∗∗*^	1	·
Tryptase	−0.009	−0.106	0.176^*∗∗*^	0.188^*∗∗*^	0.302^*∗∗*^	0.458^*∗∗*^	1
TNF-*α*	−0.052	−0.173^*∗*^	0.057	0.049	0.08	0.085	0.212^*∗∗*^

IgE: immunoglobulin E; hs-CRP: hypersensitivity C-reactive protein; IL-4: interleukin-4; IL-6: interleukin-6; IL-10: interleukin-10; Foxp3+: forkhead/winged helix transcription factor 3+; TNF-*α*: tumor necrosis factor.

^*∗*^Data on all subjects without missing values for all of these variables. ^*∗*^ *P* < 0.05. ^*∗∗*^ *P* < 0.01.

**Table 4 tab4:** Spearman correlation coefficients between inflammatory markers and cardiovascular factors in normal glucose group.

	IgE	CRP	IL-4	IL-6	IL-10	Foxp3	Tryptase	TNF-*α*
TC	−0.087	0.007	0.038	−0.083	−0.031	−0.016	0.138^*∗*^	0.163^*∗*^
TG	0.020	0.122	0.048	0.023	0.073	0.052	0.077	0.111
HDL-c	0.017	−0.146^*∗*^	−0.085	−0.136^*∗*^	−0.031	0.050	0.065	0.082
LDL-c	−0.043	−0.001	0.089	−0.001	−0.026	−0.027	0.115	0.111
HOMA-IR	0.012	0.102	0.093	−0.003	0.007	−0.114	−0.085	−0.068
HOMA-*β*	0.030	0.092	−0.006	0.053	−0.055	−0.047	0.014	−0.117
BMI	0.037	0.001	−0.006	−0.040	0.026	0.057	0.028	0.114
WHR	−0.038	−0.042	0.146^*∗*^	0.106	0.108	0.126	0.095	0.085
SBP	0.091	0.024	0.000	−0.071	−0.003	0.041	0.072	0.054
DBP	0.132	0.043	0.045	−0.024	−0.045	0.026	−0.028	0.020

TC: total cholesterol; TG: triglyceride; LDL-c: low-density lipoprotein cholesterol; HDL-c: high-density lipoprotein cholesterol; HOMA-IR: homeostasis model assessment-insulin resistance; HOMA-*β*: homeostasis model assessment-*β* cell function; BMI: body mass index; WHR: waist hip ratio; SBP: systolic blood pressure; DBP: diastolic blood pressure; IgE: immunoglobulin E; hs-CRP: hypersensitivity C-reactive protein; IL-4: interleukin-4; IL-6: interleukin-6; IL-10: interleukin-10; Foxp3+: forkhead/winged helix transcription factor 3+; TNF-*α*: tumor necrosis factor.

^*∗*^Data on all subjects without missing values for all of these variables. ^*∗*^ *P* < 0.05.

**Table 5:** ORs of prediabetes and T2DM according to the inflammatory markers.

	OR (95% CI)					
	PDG versus NGG	*P*	DMG versus NGG	*P*	DMG versus PDG	*P*
IgE						
Model 1 (unadjusted)^†^	1.34 (0.89–2.03)	0.166	3.05 (1.93–4.84)	**<0.001**	2.28 (1.45–3.28)	**<0.001**
Model 2 (age and sex)^‡^	1.35 (0.89–2.65)	0.156	3.11 (1.95–4.95)	**<0.001**	2.29 (1.46–3.59)	**<0.001**
Model 3 (multivariable)^§^	1.41 (0.89–2.24)	0.147	3.51 (1.96–6.29)	**<0.001**	2.55 (1.52–4.25)	**<0.001**
Model 4 (multivariable + CRP)	1.36 (0.85–2.19)	0.198	3.79 (1.89–7.73)	**<0.001**	2.58 (1.49–4.46)	**0.001**
Model 5 (multivariable + tryptase)	1.40 (0.88–2.23)	0.156	3.48 (1.94–6.25)	**<0.001**	2.52 (1.51–4.23)	**<0.001**
Model 6 (multivariable + IL-6)	1.40 (0.88–2.22)	0.158	3.51 (1.96–6.29)	**<0.001**	2.54 (1.52–4.25)	**<0.001**
Model 7 (multivariable + TNF-*α*)	1.42 (0.89–2.89)	0.146	3.49 (1.95–6.26)	**<0.001**	2.55 (1.52–4.25)	**<0.001**
Model 8 (multivariable + IL-4)	1.32 (0.83–2.11)	0.244	3.44 (1.91–6.18)	**<0.001**	2.55 (1.53–4.26)	**<0.001**
Model 9 (multivariable + IL-10)	1.30 (1.81–2.07)	0.283	2.72 (1.45–5.10)	**0.002**	2.33 (1.38–3.93)	**0.002**
Model 10 (multivariable + Foxp3)	1.39 (0.87–2.21)	0.167	3.46 (1.92–6.23)	**<0.001**	2.57 (1.54–4.30)	**<0.001**
CRP						
Model 1 (unadjusted)^†^	2.35 (1.57–3.52)	**<0.001**	11.76 (6.90–20.03)	**<0.001**	5.01 (3.00–8.37)	**<0.001**
Model 2 (age and sex)^‡^	2.32 (1.55–3.49)	**<0.001**	11.70 (6.84–20.02)	**<0.001**	5.11 (3.04–8.57)	**<0.001**
Model 3 (multivariable)^§^	2.34 (1.49–3.67)	**<0.001**	16.14 (7.99–32.59)	**<0.001**	5.73 (3.19–10.29)	**<0.001**
Model 4 (multivariable + IgE)	2.31 (1.47–3.64)	**<0.001**	17.23 (8.17–36.33)	**<0.001**	5.81 (3.19–10.59)	**<0.001**
Model 5 (multivariable + tryptase)	2.31 (1.47–3.63)	**<0.001**	16.17 (7.99–32.70)	**<0.001**	5.81 (3.23–10.48)	**<0.001**
Model 6 (multivariable + IL-6)	2.34 (1.49–3.68)	**<0.001**	16.30 (7.99–32.59)	**<0.001**	5.79 (3.21–10.43)	**<0.001**
Model 7 (multivariable + TNF-*α*)	2.36 (1.50–3.72)	**<0.001**	17.30 (8.43–35.52)	**<0.001**	5.75 (3.20–10.34)	**<0.001**
Model 8 (multivariable + IL-4)	2.23 (1.41–3.51)	**0.001**	17.34 (8.39–35.86)	**<0.001**	5.87 (3.24–10.61)	**<0.001**
Model 9 (multivariable + IL-10)	2.25 (1.42–3.54)	**0.001**	17.35 (8.24–35.74)	**<0.001**	5.79 (3.16–10.59)	**<0.001**
Model 10 (multivariable + Foxp3)	2.30 (1.46–3.62)	**<0.001**	16.24 (7.99–33.02)	**<0.001**	5.93 (3.29–10.72)	**<0.001**
Tryptase						
Model 1 (unadjusted)^†^	1.53 (1.01–2.32)	**0.044**	1.15 (0.70–1.88)	0.588	0.75 (0.46–1.21)	0.237
Model 2 (age and sex)^‡^	1.55 (0.99–2.29)	0.055	1.21 (0.68–1.85)	0.653	0.75 (0.46–1.21)	0.236
Model 3 (multivariable)^§^	1.57 (0.99–2.50)	0.057	1.35 (0.73–2.47)	0.336	0.75 (0.44–1.29)	0.305
Model 4 (multivariable + IgE)	1.56 (0.98–2.45)	0.06	1.32 (0.66–2.36)	0224	0.78 (0.45–1.36)	0.485
Model 5 (multivariable + CRP)	1.52 (0.95–2.44)	0.082	1.34 (0.66–2.73)	0.417	0.71 (0.40–1.25)	0.235
Model 6 (multivariable + IL-6)	1.22 (0.74–2.00)	0.436	1.39 (0.74–2.61)	0.306	0.83 (0.47–1.47)	0.527
Model 7 (multivariable + TNF-*α*)	1.47 (0.90–2.38)	0.126	1.28 (0.68–2.41)	0.440	0.71 (0.40–1.27)	0.246
Model 8 (multivariable + IL-4)	1.63 (1.02–2.61)	**0.041**	1.26 (0.67–2.30)	0.484	0.73 (0.42–1.26)	0.263
Model 9 (multivariable + IL-10)	1.36 (0.84–2.19)	0.213	0.93 (0.47–1.84)	0.841	0.64 (0.37–1.12)	0.117
Model 10 (multivariable + Foxp3)	1.50 (0.92–2.44)	0.107	1.21 (0.58–2.51)	0.612	0.77 (0.41–1.43)	0.399
IL-6						
Model 1 (unadjusted)^†^	1.37 (0.91–2.08)	0.136	0.85 (0.51–1.42)	0.534	0.62 (0.37–1.03)	0.062
Model 2 (age and sex)^‡^	1.36 (0.90–2.07)	0.146	0.86 (0.51–1.44)	0.560	0.62 (0.37–1.03)	0.062
Model 3 (multivariable)^§^	1.37 (0.86–2.19)	0.188	0.97 (0.51–1.85)	0.922	0.68 (0.39–1.18)	0.169
Model 4 (multivariable + IgE)	1.36 (0.85–2.18)	0.202	1.01 (0.52–1.96)	0.985	0.68 (0.40–1.20)	0.188
Model 5 (multivariable + CRP)	1.37 (0.85–2.22)	0.193	0.99 (0.46–2.11)	0.977	0.64 (0.36–1.18)	0.159
Model 6 (multivariable + tryptase)	1.22 (0.74–2.00)	0.436	0.88 (0.45–1.73)	0.720	0.72 (0.40–1.29)	0.264
Model 7 (multivariable + TNF-*α*)	1.41 (0.88–2.27)	0.157	0.95 (0.50–1.82)	0.876	0.67 (0.38–1.17)	0.161
Model 8 (multivariable + IL-4)	1.25 (0.77–2.02)	0.374	0.85 (0.43–1.65)	0.624	0.62 (0.35–1.12)	0.111
Model 9 (multivariable + IL-10)	1.19 (0.73–1.93)	0.483	0.68 (0.33–1.39)	0.287	0.56 (0.31–1.00)	**0.05**
Model 10 (multivariable + Foxp3)	0.68 (0.39–1.20)	0.183	0.91 (0.47–1.75)	0.769	0.68 (0.39–1.20)	0.183
TNF-*α*						
Model 1 (unadjusted)^†^	1.29 (0.85–2.97)	0.239	1.24 (0.76–2.02)	0.390	0.96 (0.59–1.53)	0.866
Model 2 (age and sex)^‡^	1.31 (0.85–2.00)	0.218	1.26 (0.77–2.07)	0.358	0.96 (0.59–1.55)	0.867
Model 3 (multivariable)^§^	1.43 (0.89–2.29)	0.140	1.29 (0.70–2.38)	0.420	1.04 (0.60–1.81)	0.883
Model 4 (multivariable + IgE)	1.42 (0.89–2.89)	0.146	1.26 (0.67–2.38)	0.480	1.04 (0.59–1.83)	0.885
Model 5 (multivariable + CRP)	1.46 (0.90–2.37)	0.127	1.83 (0.87–3.85)	0.111	0.94 (0.52–1.68)	0.829
Model 6 (multivariable + tryptase)	1.27 (0.77–2.09)	0.347	1.20 (0.63–2.28)	0.572	1.19 (0.66–2.15)	0.570
Model 7 (multivariable + IL-6)	1.41 (0.88–2.27)	0.157	1.29 (0.70–2.40)	0.414	1.10 (0.63–1.91)	0.751
Model 8 (multivariable + IL-4)	1.35 (0.84–2.18)	0.218	1.20 (0.64–2.25)	0.568	1.02 (0.58–1.78)	0.944
Model 9 (multivariable + IL-10)	1.27 (0.77–2.05)	0.352	1.24 (0.56–2.14)	0.652	0.89 (0.51–1.58)	0.694
Model 10 (multivariable + Foxp3)	1.06 (0.61–1.85)	0.840	1.24 (0.66–2.30)	0.504	1.06 (0.61–1.85)	0.840
IL-4						
Model 1 (unadjusted)^†^	1.90 (1.27–2.86)	**0.002**	2.26 (1.42–3.59)	**0.001**	1.19 (0.76–1.85)	0.456
Model 2 (age and sex)^‡^	1.93 (1.28–2.92)	**0.002**	2.27 (1.41–3.63)	**0.001**	1.19 (0.76–1.86)	0.457
Model 3 (multivariable)^§^	1.73 (1.09–2.75)	**0.020**	2.06 (1.15–3.68)	**0.015**	1.13 (0.68–1.87)	0.649
Model 4 (multivariable + IgE)	1.67 (1.05–2.66)	**0.030**	1.98 (1.10–3.58)	**0.023**	1.14 (0.68–1.90)	0.630
Model 5 (multivariable + CRP)	1.58 (1.00–2.52)	0.057	2.47 (1.22–4.98)	**0.012**	0.86 (0.50–1.50)	0.601
Model 6 (multivariable + tryptase)	1.53 (1.02–2.61)	**0.041**	2.01 (1.02–3.60)	**0.019**	1.18 (0.71–1.99)	0.522
Model 7 (multivariable + IL-6)	1.67 (1.04–2.66)	**0.033**	2.11 (1.17–3.80)	**0.013**	1.29 (0.76–2.21)	0.350
Model 8 (multivariable + TNF-*α*)	1.68 (1.06–2.67)	**0.026**	2.03 (1.13–3.63)	**0.017**	1.12 (0.67–1.87)	0.663
Model 9 (multivariable + IL-10)	1.46 (0.90–2.36)	0.125	1.58 (0.84–2.97)	0.158	0.88 (0.52–1.51)	0.645
Model 10 (multivariable + Foxp3)	1.68 (1.05–2.69)	**0.031**	1.94 (1.10–3.53)	**0.023**	1.15 (0.69–1.92)	0.601
IL-10						
Model 1 (unadjusted)^†^	1.99 (1.32–2.99)	**0.001**	5.76 (3.57–9.29)	**<0.001**	2.90 (1.84–4.58)	**<0.001**
Model 2 (age and sex)^‡^	1.03 (1.00–1.06)	0.060	6.07 (3.72–9.91)	**<0.001**	2.92 (1.84–4.62)	**<0.001**
Model 3 (multivariable)^§^	2.17 (1.37–3.46)	**0.001**	8.61 (4.51–16.41)	**<0.001**	3.03 (1.79–5.12)	**<0.001**
Model 4 (multivariable + IgE)	2.11 (1.32–3.37)	**0.002**	7.56 (3.92–14.57)	**<0.001**	2.82 (1.65–4.81)	**<0.001**
Model 5 (multivariable + CRP)	2.07 (1.29–3.31)	**0.002**	7.33 (3.27–13.68)	**<0.001**	3.06 (1.73–5.39)	**<0.001**
Model 6 (multivariable + tryptase)	2.05 (1.28–3.29)	**0.003**	8.70 (4.52–16.73)	**<0.001**	3.21 (1.18–5.48)	**<0.001**
Model 7 (multivariable + IL-6)	2.11 (1.32–3.38)	**0.002**	8.79 (4.12–16.04)	**<0.001**	3.28 (1.92–5.60)	**<0.001**
Model 8 (multivariable + TNF-*α*)	2.10 (1.31–3.36)	**0.002**	8.66 (3.99–15.86)	**<0.001**	3.07 (1.81–5.22)	**<0.001**
Model 9 (multivariable + IL-4)	1.98 (1.23–3.20)	**0.005**	8.86 (4.11–15.06)	**<0.001**	3.11 (1.81–5.33)	**<0.001**
Model 10 (multivariable + Foxp3)	2.13 (1.33–3.41)	**0.002**	8.62 (4.50–16.49)	**<0.001**	3.04 (1.79–5.13)	**<0.001**
Foxp3+						
Model 1 (unadjusted)^†^	1.35 (0.89–2.06)	0.162	1.10 (0.67–1.81)	0.702	0.82 (0.50–1.33)	0.415
Model 2 (age and sex)^‡^	1.33 (0.87–2.03)	0.190	1.07 (0.65–1.77)	0.788	0.81 (0.50–1.33)	0.410
Model 3 (multivariable)^§^	1.31 (0.81–2.17)	0.270	1.69 (0.91–3.12)	0.097	0.90 (0.52–1.57)	0.720
Model 4 (multivariable + IgE)	1.35 (0.84–2.17)	0.211	1.61 (0.85–3.04)	0.148	0.84 (0.48–1.48)	0.551
Model 5 (multivariable + CRP)	1.19 (0.73–1.95)	0.478	150 (0.65–3.24)	0.241	0.73 (0.40–1.31)	0.293
Model 6 (multivariable + tryptase)	1.17 (0.71–1.93)	0.544	1.61 (0.85–3.04)	0.144	1.01 (0.56–1.83)	0.977
Model 7 (multivariable + IL-6)	1.24 (0.76–2.02)	0.395	1.70 (0.92–3.17)	0.093	0.98 (0.56–1.73)	0.947
Model 8 (multivariable + TNF-*α*)	1.26 (0.78–2.04)	0.349	1.66 (0.89–3.07)	0.111	0.90 (0.51–1.56)	0.701
Model 9 (multivariable + IL-4)	1.17 (0.72–1.92)	0.525	1.57 (0.84–2.93)	0.161	0.88 (0.50–1.54)	0.659
Model 10 (multivariable + IL-10)	1.13 (0.69–1.86)	0.624	1.68 (0.86–3.28)	0.132	0.88 (0.50–1.55)	0.651

ORs: odd ratios; T2DM: type 2 diabetes mellitus; TC: total cholesterol; TG: triglyceride; BMI: body mass index; WHR: waist hip ratio; SBP: systolic blood pressure; DBP: diastolic blood pressure; IgE: immunoglobulin E; hs-CRP: hypersensitivity C-reactive protein; IL-4: interleukin-4; IL-6: interleukin-6; IL-10: interleukin-10; Foxp3+: forkhead/winged helix transcription factor 3+; TNF-*α*: tumor necrosis factor.

Data are OR (95% CI) unless otherwise indicated.

^†^Model 1 was not adjusted for any variable.

^‡^Model 2 was adjusted for age and sex.

^^§^^Model 3 was adjusted for age, sex, BMI, WHR, SBP, DBP, TC, TG, level of physical activity, dietary intake, alcohol intake, smoking status, presence or absence of family history of diabetes, hypertension, heart disease, stroke, and hypercholesterolemia.
