# Supplementary material for: Analysis of Inflammatory Mediators in Prediabetes and Newly Diagnosed Type 2 Diabetes Patients
Source: J Diabetes Res. 2016 Jul 5;2016:7965317. doi: 10.1155/2016/7965317 (PMC4949350; doi:10.1155/2016/7965317)
Supplement: Supplementary file 1 — Tables S1-S6 described the spearman correlation between inflammatory cytokines and the traditional cardiovascular factors and indicated that whether in the Pre-diabetes group or the type 2 diabetes mellitus group, there were significant relationships between most of the traditional cardiovascular factors and between most of inflammatory cytokines. However, there were seldom significant relationships between the traditional cardiovascular factors and inflammatory cytokines (Tables S1-S6). [file 7965317.f1.docx]

**Table S1.**

**Spearman correlation coefficients between cardiovascular factors in pre-diabetes group**

Zhen Wang, Xu-Hui Shen, Wen-Ming Feng, Guo-fen Ye, Wei Qiu, Xu-Hong Wu, Bo Li, Tian-Bao Lu, Ying He, Guo-Ping Shi

**Table S1.** **Spearman correlation coefficients between cardiovascular factors in pre-diabetes group**

|  | TC | TG | HDL-c | LDL-c | HOMA-IR | HOMA-β | BMI | WHR | SBP |
| --- | --- | --- | --- | --- | --- | --- | --- | --- | --- |
| TC | 1 |  |  |  |  |  |  |  |  |
| TG | 0.295** | 1 |  |  |  |  |  |  |  |
| HDL-c | 0.264** | -.461** | 1 |  |  |  |  |  |  |
| LDL-c | 0.830** | -0.071 | 0.228** | 1 |  |  |  |  |  |
| HOMA-IR | 0.015 | 0.027 | 0.077 | 0.009 | 1 |  |  |  |  |
| HOMA-β | -0.067 | -0.075 | 0.081 | 0.005 | -0.596** | 1 |  |  |  |
| BMI | 0.024 | 0.296** | -0.287** | 0.005 | -0.064 | -0.260** | 1 |  |  |
| WHR | -0.006 | 0.266** | -0.264** | -0.037 | -0.074 | -0.205** | 0.501** | 1 |  |
| SBP | 0.096 | 0.063 | -0.037 | 0.107 | -0.032 | -0.177** | 0.281** | 0.215** | 1 |
| DBP | 0.050 | 0.034 | -0.073 | 0.085 | -0.045 | -0.123 | 0.225** | 0.195** | 0.691** |

TC: total cholesterol; TG:triglyceride; LDL-c: low-density lipoprotein cholesterol; HDL-c: high-density lipoprotein cholesterol; HOMA-IR: Homeostasis model assessment–insulin resistance; HOMA-β:Homeostasis model assessment -β cell function; BMI: body mass index; WHR: waist hip ratio; SBP: systolic blood pressure; DBP: diastolic blood pressure.

.*Data on all subjects without missing values for all of these variables.*P ＜0.05. **P ＜0.01

**Table S2.**

**Spearman correlation coefficients between inflammatory markers in pre-diabetes group**

Zhen Wang, Xu-Hui Shen, Wen-Ming Feng, Guo-fen Ye, Wei Qiu, Xu-Hong Wu, Bo Li, Tian-Bao Lu, Ying He, Guo-Ping Shi

**Table S2.** **Spearman correlation coefficients between inflammatory markers in pre-diabetes group**

|  | **IGE** | **hs-CRP** | **IL-4** | **IL-6** | **IL-10** | **Foxp3+** | **Tryptase** |  |
| --- | --- | --- | --- | --- | --- | --- | --- | --- |
| **IGE** | | 1.000 |  |  |  |  |  |  |
| **hs-CRP** | | 0.087 | 1.000 |  |  |  |  |  |
| **IL-4** | | 0.052 | 0.135* | 1.000 |  |  |  |  |
| **IL-6** | | -0.014 | -0.037 | 0.485** | 1.000 |  |  |  |
| **IL-10** | | 0.100 | 0.147* | 0.394** | 0.500** | 1.000 |  |  |
| **Foxp3+** | | 0.011 | 0.063 | 0.255** | 0.353** | 0.271** | 1.000 |  |
| **Tryptase** | | 0.013 | -0.046 | 0.276** | 0.404** | 0.297** | 0.523** | 1.000 |
| **TNF-a** | | 0.019 | 0.059 | 0.221** | 0.117 | 0.085 | 0.151* | 0.372** |

IgE: immunoglobulin E; hs-CRP: hypersensitivity C-reactive protein; IL-4: interleukin-4; IL-6: interleukin-6; IL-10: interleukin-10; **Foxp3+:** forkhead/winged helix transcription factor 3+;TNF-a: tumor necrosis factor.

*Data on all subjects without missing values for all of these variables.*P ＜0.05. **P ＜0.01

**Table S3.**

**Spearman correlation coefficients between inflammatory markers and cardiovascular factors in pre-diabetes group**

Zhen Wang, Xu-Hui Shen, Wen-Ming Feng, Guo-fen Ye, Wei Qiu, Xu-Hong Wu, Bo Li, Tian-Bao Lu, Ying He, Guo-Ping Shi

**Table S3.** **Spearman correlation coefficients between inflammatory markers and cardiovascular factors in pre-diabetes group**

|  | IGE | CRP | IL-4 | IL-6 | IL-10 | Foxp3 | Tryptase | TNF-a |
| --- | --- | --- | --- | --- | --- | --- | --- | --- |
| TC | 0.000 | 0.133 | 0.146* | -0.067 | 0.084 | -0.064 | -0.109 | -0.018 |
| TG | 0.039 | 0.062 | 0.023 | -0.033 | 0.007 | 0.106 | -0.074 | -0.157* |
| HDL-c | -0.108 | -0.061 | 0.012 | -0.033 | -0.026 | -0.158* | -0.013 | 0.152* |
| LDL-c | 0.016 | 0.122 | 0.120 | -0.032 | 0.142* | -0.050 | -0.076 | 0.005 |
| HOMA-IR | -0.047 | 0.119 | -0.130 | -0.092 | 0.059 | 0.019 | 0.028 | -0.052 |
| HOMA-β | -0.091 | 0.067 | -0.001 | 0.076 | 0.089 | 0.056 | 0.080 | 0.047 |
| BMI | 0.010 | 0.007 | -0.002 | -0.034 | 0.052 | -0.050 | -0.071 | -0.051 |
| WHR | 0.076 | 0.011 | 0.086 | -0.049 | 0.063 | -0.083 | -0.172* | -0.207** |
| SBP | -0.035 | 0.067 | -0.060 | -0.133 | -0.018 | -0.200** | -0.249** | -0.143* |
| DBP | -0.004 | 0.121 | -0.060 | -0.116 | -0.007 | -0.114 | -0.237** | -0.073 |

TC: total cholesterol; TG:triglyceride; LDL-c: low-density lipoprotein cholesterol; HDL-c: high-density lipoprotein cholesterol; HOMA-IR: Homeostasis model assessment–insulin resistance; HOMA-β:Homeostasis model assessment -β cell function; BMI: body mass index; WHR: waist hip ratio; SBP: systolic blood pressure; DBP: diastolic blood pressure. IgE: immunoglobulin E; hs-CRP: hypersensitivity C-reactive protein; IL-4: interleukin-4; IL-6: interleukin-6; IL-10: interleukin-10; **Foxp3+:** forkhead/winged helix transcription factor 3+;TNF-a: tumor necrosis factor.

*Data on all subjects without missing values for all of these variables.*P ＜0.05. **P ＜ 0.01

**Table S4.**

**Spearman correlation coefficients between cardiovascular factors in T2DM group**

Zhen Wang, Xu-Hui Shen, Wen-Ming Feng, Guo-fen Ye, Wei Qiu, Xu-Hong Wu, Bo Li, Tian-Bao Lu, Ying He, Guo-Ping Shi

**Table S4.** **Spearman correlation coefficients between cardiovascular factors in T2DM group**

|  | TC | TG | HDL-c | LDL-c | HOMA-IR | HOMA-β | BMI | WHR | SBP |
| --- | --- | --- | --- | --- | --- | --- | --- | --- | --- |
| TC | 1 |  |  |  |  |  |  |  |  |
| TG | 0.449** | 1 |  |  |  |  |  |  |  |
| HDL-c | 0.099 | -0.524** | 1 |  |  |  |  |  |  |
| LDL-c | 0.770** | -0.024 | 0.270** | 1 |  |  |  |  |  |
| HOMA-IR | -0.049 | 0.000 | 0.062 | -0.073 | 1 |  |  |  |  |
| HOMA-β | -0.057 | -0.116 | 0.095 | -0.004 | -0.618** | 1 |  |  |  |
| BMI | 0.129 | 0.241** | -0.031 | 0.110 | -0.137 | -0.146 | 1 |  |  |
| WHR | 0.061 | 0.117 | -0.021 | -0.004 | 0.000 | -0.085 | 0.245** | 1 |  |
| SBP | 0.279** | 0.175 | .188* | 0.161 | 0.140 | -0.089 | 0.357** | 0.272** | 1 |
| DBP | 0.112 | -0.025 | 0.087 | 0.177(*) | -0.071 | -0.078 | 0.200* | -0.016 | 0.223* |

TC: total cholesterol; TG:triglyceride; LDL-c: low-density lipoprotein cholesterol; HDL-c: high-density lipoprotein cholesterol; HOMA-IR: Homeostasis model assessment–insulin resistance; HOMA-β:Homeostasis model assessment -β cell function; BMI: body mass index; WHR: waist hip ratio; SBP: systolic blood pressure; DBP: diastolic blood pressure.

*Data on all subjects without missing values for all of these variables.*P ＜0.05. **P ＜0.01

**Table S5.**

**Spearman correlation coefficients between inflammatory markers in T2DM group**

Zhen Wang, Xu-Hui Shen, Wen-Ming Feng, Guo-fen Ye, Wei Qiu, Xu-Hong Wu, Bo Li, Tian-Bao Lu, Ying He, Guo-Ping Shi

**Table S5. Spearman correlation coefficients between inflammatory markers in T2DM group**

|  | **IGE** | **CRP** | **IL-4** | **IL-6** | **IL-10** | **Foxp3** | **Tryptase** |
| --- | --- | --- | --- | --- | --- | --- | --- |
| **IGE** | 1.000 |  |  |  |  |  |  |
| **CRP** | -0.055 | 1.000 | . |  |  |  |  |
| **IL-4** | -0.074 | 0.232** | 1.000 |  |  |  |  |
| **IL-6** | -0.092 | 0.009 | 0.244** | 1.000 |  |  |  |
| **IL-10** | 0.024 | 0.098 | 0.067 | 0.209* | 1.000 |  |  |
| **Foxp3** | 0.012 | 0.028 | 0.011 | 0.282** | -0.052 | 1.000 |  |
| **Tryptase** | -0.023 | -0.021 | 0.096 | 0.350** | 0.071 | 0.549** | 1.000 |
| **TNF-a** | -0.016 | -0.075 | 0.070 | 0.146 | 0.077 | 0.271** | 0.409** |

IgE: immunoglobulin E; hs-CRP: hypersensitivity C-reactive protein; IL-4: interleukin-4; IL-6: interleukin-6; IL-10: interleukin-10; **Foxp3+:** forkhead/winged helix transcription factor 3+;TNF-a: tumor necrosis factor.

*Data on all subjects without missing values for all of these variables.*P ＜0.05. **P ＜ 0.01

**Table S6.**

**Spearman correlation coefficients between inflammatory markers and cardiovascular factors in T2DM group**

Zhen Wang, Xu-Hui Shen, Wen-Ming Feng, Guo-fen Ye, Wei Qiu, Xu-Hong Wu, Bo Li, Tian-Bao Lu, Ying He, Guo-Ping Shi

**Correlati**

**Table S6. Spearman correlation coefficients between inflammatory markers and cardiovascular factors in T2DM group**

|  | IGE | CRP | IL-4 | IL-6 | IL-10 | Foxp3+ | Tryptase | TNF-a |
| --- | --- | --- | --- | --- | --- | --- | --- | --- |
| TC | 0.032 | -0.017 | -0.058 | 0.028 | -0.155 | 0.063 | 0.066 | -0.036 |
| TG | -0.044 | 0.005 | -0.131 | -0.073 | -0.161 | -0.147 | -0.246** | -0.042 |
| HDL-c | 0.068 | 0.081 | 0.014 | 0.067 | 0.128 | 0.191* | 0.275** | 0.106 |
| LDL- c | 0.009 | -0.085 | -0.080 | 0.015 | -0.069 | 0.198* | 0.228* | 0.085 |
| HOMA-IR | -0.081 | 0.147 | -0.090 | 0.100 | -0.006 | 0.091 | 0.040 | -0.027 |
| HOMA-β | -0.003 | 0.163 | -0.068 | 0.097 | 0.145 | 0.194* | 0.067 | 0.041 |
| BMI | -0.129 | -0.060 | -0.111 | -0.033 | -0.093 | 0.076 | 0.052 | 0.023 |
| WHR | 0.011 | 0.065 | -0.053 | -0.123 | -0.13 | -0.179* | -0.143 | -0.030 |
| SBP | 0.023 | 0.163 | -0.030 | 0.068 | 0.002 | 0.001 | 0.040 | -0.119 |
| DBP | 0.059 | -0.100 | -0.053 | 0.140 | 0.181* | 0.089 | 0.152 | 0.097 |

TC: total cholesterol; TG:triglyceride; LDL-c: low-density lipoprotein cholesterol; HDL-c: high-density lipoprotein cholesterol; HOMA-IR: Homeostasis model assessment–insulin resistance; HOMA-β:Homeostasis model assessment -β cell function; BMI: body mass index; WHR: waist hip ratio; SBP: systolic blood pressure; DBP: diastolic blood pressure. IgE: immunoglobulin E; hs-CRP: hypersensitivity C-reactive protein; IL-4: interleukin-4; IL-6: interleukin-6; IL-10: interleukin-10; **Foxp3+:** forkhead/winged helix transcription factor 3+;TNF-a: tumor necrosis factor.

*Data on all subjects without missing values for all of these variables.*P ＜0.05. **P ＜ 0.01
